# Blastic Plasmacytoid Dendritic Cell Neoplasm

**DOI:** 10.18295/squmj.6.2024.029

**Published:** 2024-08-29

**Authors:** Dmitry V. Kravchenko, Dmitry A. Zinovkin, Denis A. Davydov, Pavel G. Kisialeu, Pavel A. Kopschaj, Oleg Savchenko, Mariya Savchenko, Maryna V. Barauniova, Anna S. Portyanko, Md Zahidul Islam Pranjol

**Affiliations:** 1 Department of Hematology. Republican Research Center for Radiation Medicine and Human Ecology, Gomel, Belarus; 2 Department of Pathology, Gomel State Medical University, Gomel, Belarus; 3 Laboratory of Morphology, Molecular & Cellular Biology, N.N.Alexandrov National Cancer Centre of Belarus, Minsk, Belarus; 4 Republican Molecular Genetic Laboratory of Carcinogenesis, N.N.Alexandrov National Cancer Centre of Belarus, Minsk, Belarus; 5 Department of Thoracic Oncology, N.N.Alexandrov National Cancer Centre of Belarus, Minsk, Belarus; 6 Department of Oncological (Chemotherapeutic) Day Care, N.N.Alexandrov National Cancer Centre of Belarus, Minsk, Belarus; 7 School of Life Sciences, University of Sussex, Brighton, UK

A 46-year-old female patient was admitted to the Department of Hematology of a research centre in Gomel, Belarus, in 2022 with marked general weakness and papular rashes on the skin with a maroon tinge [Figure 1A]. Skin incisional and breast core biopsies were performed. The dermis and subcutaneous fat exhibited a diffuse, relatively uniform infiltrate without apparent involvement of the epidermis or adnexa. The cells were small to medium-sized with round nuclei devoid of conspicuous nucleoli [Figure 1B]. A positron emission tomography-computed tomography (PET-CT) scan showed numerous areas of increased 18F-FDG uptake distributed throughout the dermis and subcutaneous tissues of the trunk and extremities, with the largest concentrations observed in the breast tissues (d = 4.5 cm, SUVmax = 6.5). Hypermetabolic substrates were also noticed in left inguinal lymph node [Figure 1C]. Immunohistochemical analysis revealed that tumour cells expressed CD45, CD43, CD56, TdT, CD4, bcl2 and bcl6 [Figure 2]. The proliferation rate (Ki-67) was approximately 30–40%. Tumour cells were negative for CD2, CD3, CD5, CD7, CD8, CD15, CD20, CD21, CD30, CD34, CD68, CD117, ALK, PD1, EBV, cyclinD1, granzyme B and perforin. The diagnosis of blastic plasmacytoid dendritic cell neoplasm (BPDCN) was confirmed.

Given the similarity of BPDCN cells to lymphoid cells, treatment with the CHOP-related protocol (DA-EPOCH) was initiated in February 2022. A positive response was observed at the end of the course; the majority of tumour masses in soft tissues were no longer reliably detected, and the remaining masses had decreased in size. After 5 courses, hematopoietic stem cells were mobilised and collected in August 2022 for subsequent autologous transplantation. One month later, the patient’s condition drastically deteriorated; she experienced weakness, spinal pain, skin rashes and blastocytosis [Figure 3]. In response to the relapse of BPDCN post-DA-EPOCH protocol, a ‘7 + 3’ chemotherapy course was administered during October–November 2022, targeting the myeloid features of the tumour cells. However, this did not achieve a second remission and the patient developed bacterial pneumonia by the end of the course. Considering this complication, monotherapy with azacytidine at dose 75 mg/m^2^ (150 mg/day subcutaneously, days 1–7) was initiated in January 2023 to achieve remission and treat the pneumonia. Antimicrobial therapy was also administered for the bacterial pneumonia; 10 days after the start of the azacytidine course, a positive response was observed. There were stable levels of haemoglobin and platelet count without the need for blood transfusions, absence of agranulocytosis and blast cells in the complete blood count and resolution of pneumonia. The clinical–laboratory remission lasted for over 1 month.

As the criteria for defining complete remission in BPDCN remains undetermined, we adopted the remission criteria used for acute myeloid leukemias (AML). These criteria include: (1) less than 5% blasts in the bone marrow with a count of at least 200 nucleated cells; (2) absence of blasts in the peripheral blood; (3) an absolute neutrophil count exceeding 1,000/μL; and (4) a platelet count exceeding 100 × 10^9^/L.

Patient consent for publication was obtained.

## Comment

BPDCN is a rare (under 0.5%), clinically aggressive haematological malignancy originating from precursors of plasmacytoid dendritic cells. This malignancy predominantly affects men over 60 years old.^1^ However, as illustrated in the current case, BPDCN can also manifest in young and paediatric patients. The disease typically involves multiple sites, commonly affecting the skin, bone marrow, lymph nodes and peripheral blood. In approximately 90% of cases, BPDCN initially presents with cutaneous involvement, which tends to persist as the disease progresses to involve multiple organs, leading to the patient's death. Current hypotheses suggest that the skin may initially serve as a ‘shelter’ organ, delaying or restricting BPDCN progression.^2^

The diagnosis of BPDCN relies on the expression pattern of specific markers such as CD4, CD56 and the absence of B- and T-lymphocytes, NK cells and myeloid or monocytic cells. Additional markers such as CD123, CD303 and TCL1, specific to plasmacytoid dendritic cells, aid in confirming the diagnosis.^3^ However, not all cases express these markers uniformly, complicating diagnosis, particularly in smaller histopathology laboratories in low-income countries where recommended immunohistochemical markers may not be routinely utilised. Therefore, BPDCN diagnosis often involves a process of exclusion based on clinical presentation, histopathological findings including multiple skin nodules, PET-CT disease-specific changes and the immunophenotypic profile of malignant cells expressing CD4, CD56, CD43, Tdt and CD45 while lacking expression of other lineage markers such as B- and T-lymphocytes, NK-cells, monocytes and myeloid lineage markers. Due to resource limitations, markers such as CD303, TCL1A, CD2AP, SPIB and TCF4 were not assessed in the current case.^4^

Systemic chemotherapy regimens commonly used in AML management are also employed in treating patients with BPDCN. Various chemotherapy protocols have shown varying levels of clinical response in patients with BPDCN.^5^ For instance, a study by Yun *et al*. examined treatment outcomes in 42 patients with BPDCN, revealing that the hyper-CVAD regimen yielded a higher complete response rate compared to CHOP-based regimens or Tagraxofusp (91% versus 50% versus 50%), although statistical significance was not reached. Currently, effective chemotherapeutic strategies specific to BPDCN remain limited, with a reported 5-year overall survival rate exceeding 20%.^6^

In published literature, both venetoclax and azacitidine have shown short response durations, necessitating combination with other modalities and further investigation to extend their efficacy.^7^ The current case underscores the potential of hypomethylating agents as a viable treatment option for patients with BPDCN, particularly those facing infectious complications.

## Figures and Tables

**Figure 1:**
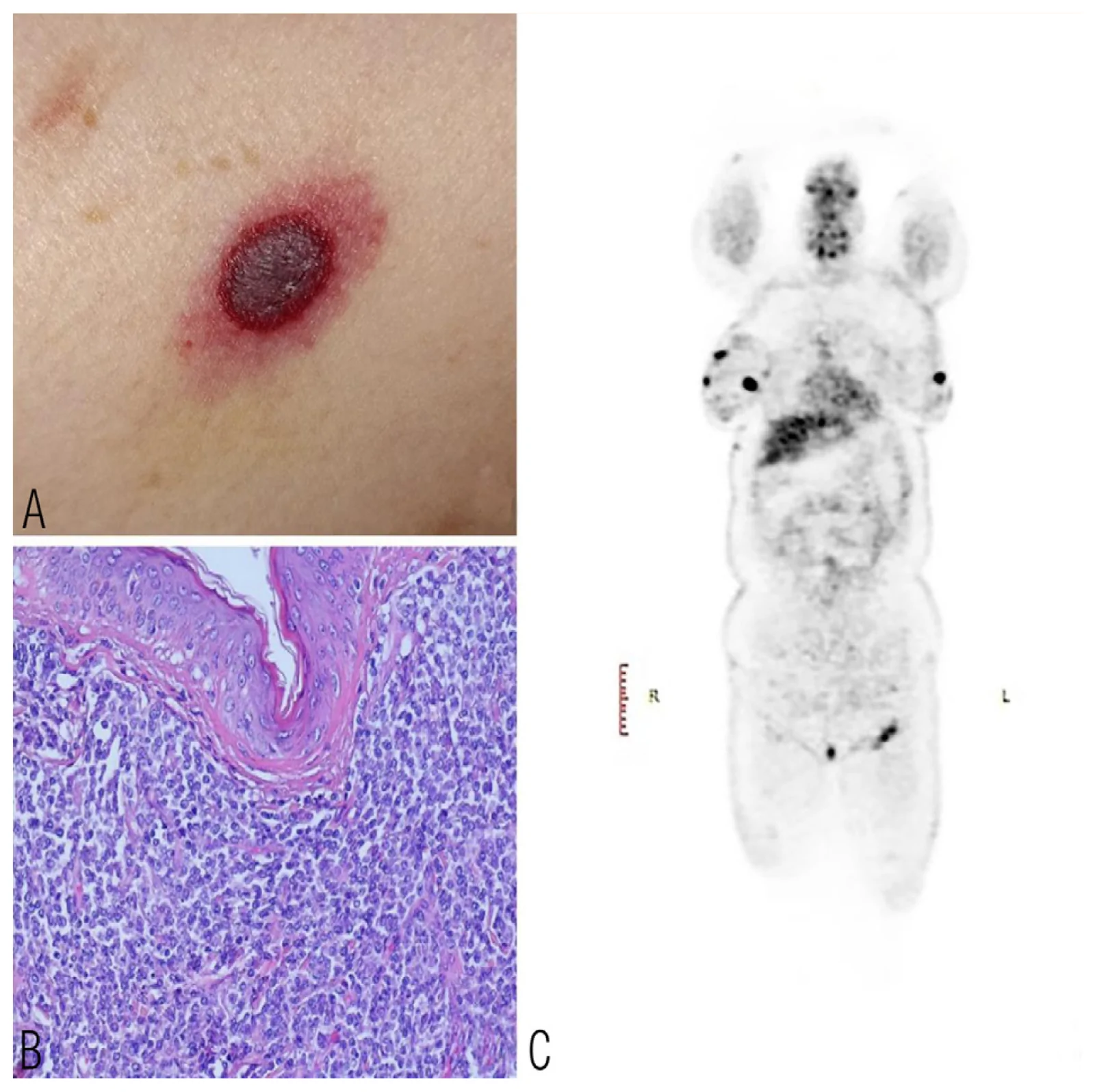
**A**: Blastic plasmacytoid dendritic cell neoplasm presenting as an erythematous papule on the skin. **B**: Haematoxylin and eosin stain at ×200 magnification showing small-to-medium-sized neoplastic blasts with fine chromatin and scanty cytoplasm. **C**: Positron emission tomography-computed tomography scan showing multiple 18F-FDG-avid foci in the breast tissues and inguinal lymph node.

**Figure 2:**
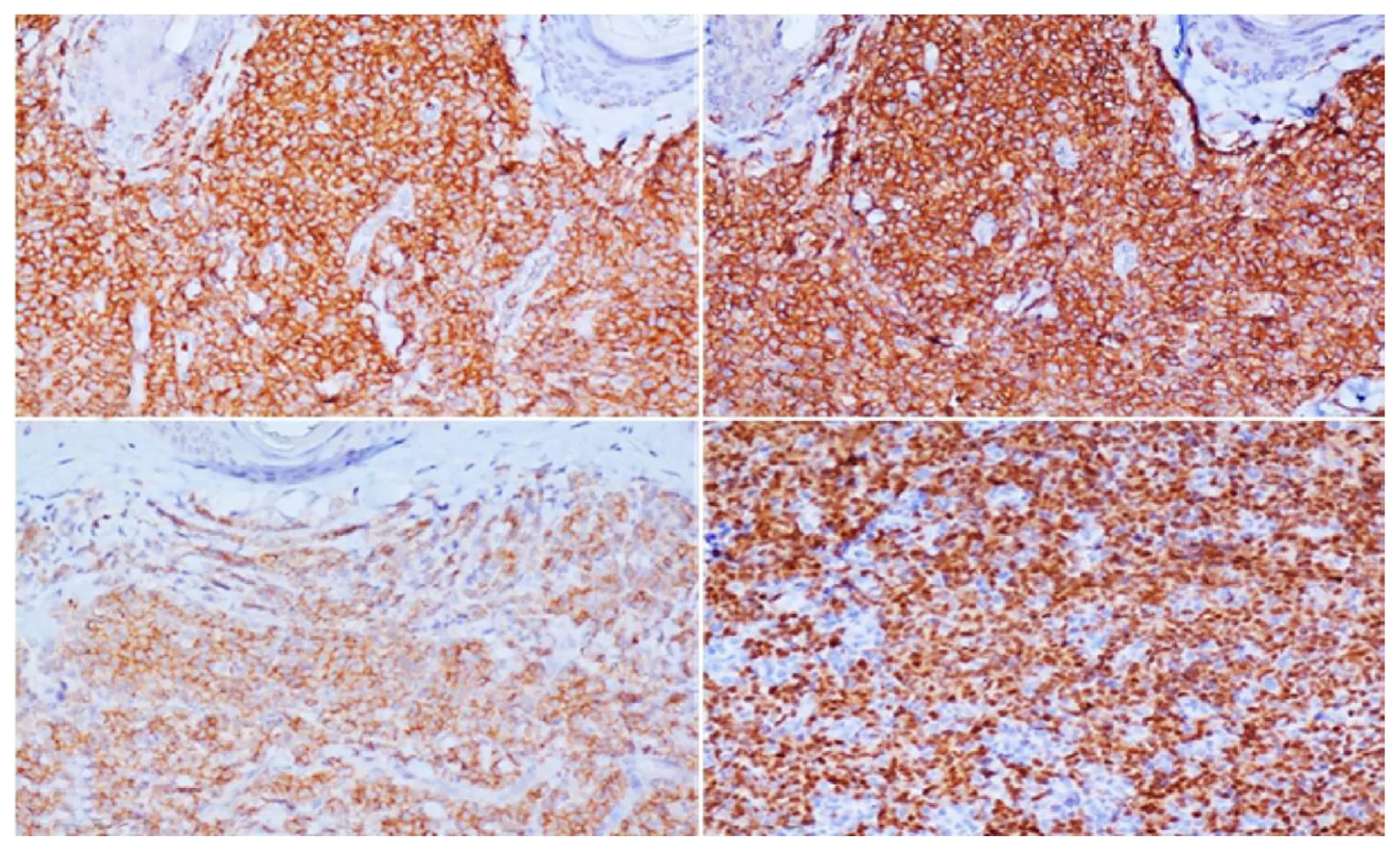
HRP-polymer-based immunohistochemistry showing strong CD45 and CD43, along with moderate CD56 expression. The majority of cells are expressing TdT. Counterstain: Meyer’s haematoxylin at magnification ×200.

**Figure 3:**
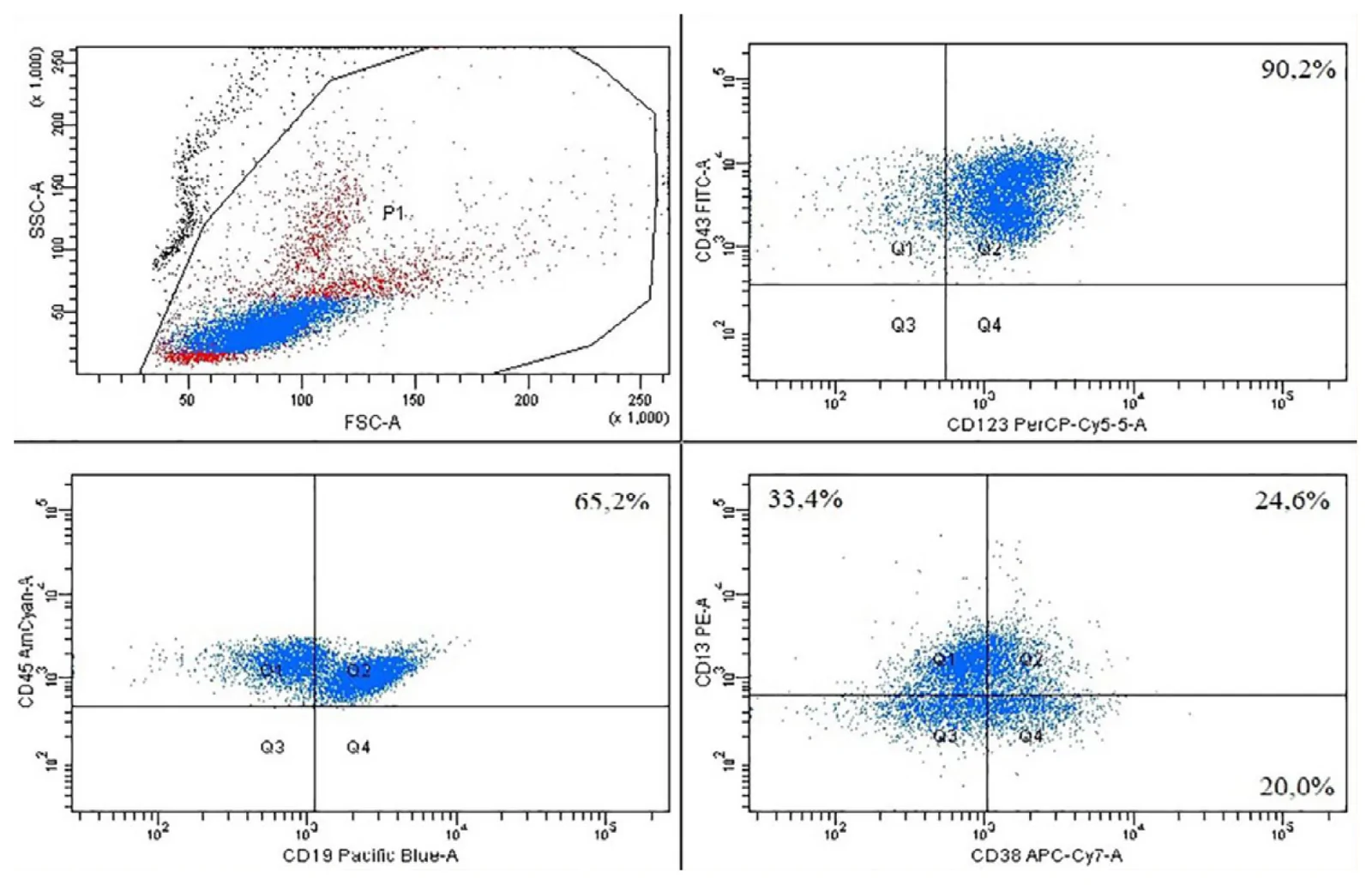
Flow cytometry of the bone marrow demonstrating hematogenous dissemination with presence of neoplastic cells in the peripheral blood.
